# Association of periconceptional or pregnancy exposure of HPV vaccination and adverse pregnancy outcomes: a systematic review and meta-analysis with trial sequential analysis

**DOI:** 10.3389/fphar.2023.1181919

**Published:** 2023-05-09

**Authors:** Xiaoli Yan, Hongyu Li, Bin Song, Ge Huang, Qing Chang, Dan Wang, Ping Yan

**Affiliations:** Department of Gynecology and Obstetrics, Southwest Hospital, Third Military Medical University (Army Medical University), Chongqing, China

**Keywords:** human papillomavirus, vaccine, pregnancy, adverse pregnancy outcomes, meta-analysis

## Abstract

**Objective:** To evaluate whether periconceptional or pregnancy exposure of human papillomavirus (HPV) vaccination would increase the risk of adverse pregnancy outcomes.

**Methods:** The PubMed, Web of Science, Embase, the Cochrane Library of clinical trials were searched from inception to March 2023. We computed relative risk (RR) and 95% confidence intervals (CIs) and prediction intervals (PIs) regarding the association between HPV vaccination in periconceptional period or during pregnancy and the risks of adverse pregnancy outcomes by using R software Version 4.1.2 and STATA Version 12.0. A trial sequential analysis (TSA) was performed with TSA v0.9.5.10 Beta software.

**Results:** Four randomized controlled trials (RCTs) and eight cohort studies were included in this meta-analysis. Analysis of RCTs showed that HPV vaccination in periconceptional period or during pregnancy did not increase the risks of spontaneous abortion (RR = 1.152, 95% CI: 0.909–1.460, 95% PI: 0.442–3.000), birth defects (RR = 1.171, 95% CI: 0.802–1.709, 95% PI: 0.320–4.342), stillbirth (RR = 1.053, 95% CI: 0.616–1.800, 95% PI: 0.318–3.540), preterm birth (RR = 0.940, 95% CI: 0.670–1.318) and ectopic pregnancy (RR = 0.807, 95% CI: 0.353–1.842, 95% PI: 0.128–5.335). In cohort studies, periconceptional or pregnancy exposures of HPV vaccine were not associated with the increased risk of spontaneous abortion (RR = 0.987, 95% CI: 0.854–1.140, 95% PI: 0.652–1.493), birth defects (RR = 0.960, 95% CI: 0.697–1.322, 95% PI: 0.371–2.480), stillbirth (RR = 1.033, 95% CI: 0.651–1.639, 95% PI: 0.052–21.064), small size for gestational age (SGA) (RR = 0.971, 95% CI: 0.873–1.081, 95% PI: 0.657–1.462) and preterm birth (RR = 0.977, 95% CI: 0.874–1.092, 95% PI: 0.651–1.444).

**Conclusion:** HPV vaccine exposures in periconceptional period or during pregnancy did not increase the risks of adverse pregnancy outcomes, including spontaneous abortion, birth defects, stillbirth, SGA, preterm birth and ectopic pregnancy.

**Systematic Review Registration:** https://www.crd.york.ac.uk/prospero/, identifier CRD42023399777.

## Introduction

Cervical cancer is the fourth most common cancer of women in the world. An estimated 570,000 new cases and 311,000 deaths were reported worldwide in 2018 (Bray et al., 2018). High-risk human papillomavirus (HPV) persistent infection is the leading cause of cervical cancer (Kjær et al., 2010). HPV vaccine, as the only vaccine to prevent cervical cancer, has been used among 72 million women worldwide since it was first approved in 2006 and has been demonstrated to be effective and safe in preventing the development of high-grade cervical cancer (Markowitz et al., 2007; Medeiros et al., 2009). Three prophylactic HPV vaccines are currently available, including bivalent vaccine (2vHPV), quadrivalent vaccine (4vHPV) and nonavalent vaccine (9vHPV), which target either two or seven high-risk HPV genotypes (Harper and DeMars, 2017). The 2vHPV and 4vHPV vaccines target HPV-16 and HPV-18, which leads to about 70% of cervical cancers worldwide, while the 9vHPV vaccine targets seven high-risk HPV genotypes (HPV-16/18/31/33/45/52/58), which causes approximately 90% of cervical cancer cases in the world (Burger et al., 2021).

The Advisory Committee on Immunization Practice has recommended routine vaccination of HPV vaccine in girls aged 11–12 years with supplementary vaccination for women under 26 years of age (Markowitz et al*.*, 2007). Therefore, large numbers of women at childbearing age may be exposed to HPV vaccination. These include those who may be unintentionally vaccinated in periconceptional period or during pregnancy, especially those who were unplanned or unrecognized pregnant (Finer and Zolna, 2016). It was reported that HPV vaccine exposure occurred during or around the time of 1.5% of pregnancies among female adolescents and young adults aged 13–27 years who received care in seven large health systems from 2007 to 2013 (Lipkind et al., 2017). In view of the lack of well controlled studies in pregnant women, fears of teratogenicity or other potentially adverse pregnancy outcome to the pregnant woman or the unborn child, such as spontaneous abortion, birth defects, preterm birth and stillbirth, have arisen among both recipients and healthcare providers (Canfell, 2015; Bonde et al., 2016; Gidengil et al., 2021). A previous study showed that the incidence of spontaneous abortion and stillbirth among women who received HPV vaccine in periconceptional period or during pregnancy was higher than that of women not vaccinated with HPV vaccine within this specific period (Moreira et al., 2016). The latest analysis suggested the peripregnancy or during-pregnancy HPV vaccine exposure was not associated with an increased risk of spontaneous abortion, preterm births, small size for gestational age (SGA) and birth defects (Kharbanda et al., 2021).

Whether HPV vaccination in the periconceptional period or during pregnancy will increase the risk of adverse maternal or infant outcomes remains largely uncertain. Although an analysis has been conducted to assess the association of periconceptional or pregnancy exposure of HPV vaccination and the risk of spontaneous abortion (Tan et al., 2019). Given the current absence of a comprehensive systematic review of HPV vaccination in the periconceptional period or during pregnancy and adverse maternal or infant outcomes (e.g., birth defects, stillbirth, small size for gestational age, preterm birth and ectopic pregnancy), we conducted a meta-analysis of all relevant clinical research evidence to further explore whether periconceptional or pregnancy exposure of HPV vaccination increased the risk of adverse pregnancy outcomes in randomized controlled trials (RCTs) and cohort studies, respectively.

## Materials and methods

This study does not require ethical approval and informed consent because it is a systematic review and meta-analysis of previously published literature and does not address ethics or patient privacy. Our study was analyzed and reported according to the Preferred Reporting Items for Systematic Reviews and Meta-Analyses (PRISMA) (Moher et al., 2009). The protocol for this meta-analysis has been registered in the PROSPERO database (CRD42023399777).

### Search strategy

We thoroughly searched the PubMed, Web of Science, Embase, and the Cochrane Library of clinical trials for all potential articles from inception to March 2023, using the following search items: (“human papillomavirus virus”, “HPV”, “human papilloma virus”, “vaccine”, “vaccination”, “vaccinated”) AND (“pregnant women,” “pregnancy,” “conception,” “parturient,” “child bearing”) AND (“preterm,” “small for gestational age,” “spontaneous abortion,” “stillbirth,” “birth defect,” “reproductive outcome,” “pregnancy outcome”). The detailed search strategy was provided in Supplementary Material S1. References within the identified articles were manually examined to identify other potentially eligible studies.

### Inclusion and exclusion criteria

Studies should meet the following inclusion criteria: 1) clinical trials or cohort studies if they contained primary data regarding pregnant women who received HPV vaccine, 2) describe the association between HPV vaccine exposures in periconceptional period or during pregnancy and the risk of adverse pregnancy outcomes, and 3) report adverse maternal or fetal outcomes (primary outcomes: spontaneous abortion and birth defects, secondary outcomes: stillbirth, small size for gestational age, preterm birth and ectopic pregnancy). Accordingly, the exclusion criteria were as follows: 1) studies with unusable or duplicate outcome data, 2) non-controlled studies, and 3) conference abstracts, reviews, case reports, and meta-analyses.

### Data extraction

Two independent researchers screened the literature and extracted all needed information from the included studies. All disagreements were resolved by discussion with a third investigator. The following information was extracted from each article using the predesigned data-collection form: name of first author, publication year, country or region, study design, study time, sample size, vaccination exposure time and vaccine type of exposure group and control group, age of the study population, duration of follow-up and outcomes.

### Risk of bias assessment

Cohort studies were assessed using the Newcastle-Ottawa scale (NOS) (Wells et al., 2014) consisting of three domains: i) selection of subjects, ii) comparability of groups, and iii) assessment of outcome. A score of 0–9 was allocated to each relevant study. While the NOS has no established thresholds, we considered the quality of each study as low (0–3 score), moderate (4–6 score), or high (7–9 score) (Chung et al., 2021). We used the modified Jadad scale to assess the quality of RCTs (Jadad et al., 1996). The evaluation criteria of the modified Jadad scale included four items: randomization, randomization concealment, double blind, and withdrawals and dropouts. The score 0–3 out of 7 was considered a low-quality study and a score of 4–7 was a high-quality study. When inconsistency exists, a third reviewer will make the final decision after verification and discussion.

### Statistical analysis

The comparison of adverse pregnancy outcomes between HPV exposure group and control group were estimated by the relative risk (RR) and their 95% confidence intervals (CIs). Heterogeneity was assessed statistically by using the Cochran’s Q test, I^2^ and Tau^2^ statistic and 95% prediction interval (PI) (Bowden et al., 2011; IntHout et al., 2016). When I^2^ ≤ 50% or *p* > 0.1, the results of the associated studies were considered to have acceptable heterogeneity, and a fixed-effects model was utilized. When I^2^ > 50% or *p* ≤ 0.1, it was considered that there was heterogeneity in the results of the included studies, and a random-effects model was selected (Higgins and Thompson, 2002). Sensitivity analysis was conducted to explore the possible sources of heterogeneity. The presence of publication bias was assessed with the Egger’s regression asymmetry test (Sterne and Egger, 2001). Statistical analyses were performed with R software Version 4.1.2 and STATA Version 12.0 (StataCorp, College Station, TX, United States).

### Trial sequential analysis

A trial sequential analysis (TSA) was performed to assess if the available evidence is up to the required information size (RIS) for robust conclusion (Wetterslev et al., 2017). For dichotomous outcomes, the trial sequential analysis was performed with TSA v0.9.5.10 Beta software (www.ctu.dk/tsa). We calculated the RIS and built O’ Brien-Fleming α-spending boundaries by using type I error of 5% and type II error of 20%, which were two-side values. If the cumulative Z-curve crossed the trial sequential monitoring boundary or RIS boundary, no further trials were considered to be needed and firm evidence was obtained.

## Results

### Literature search

**FIGURE 1:**
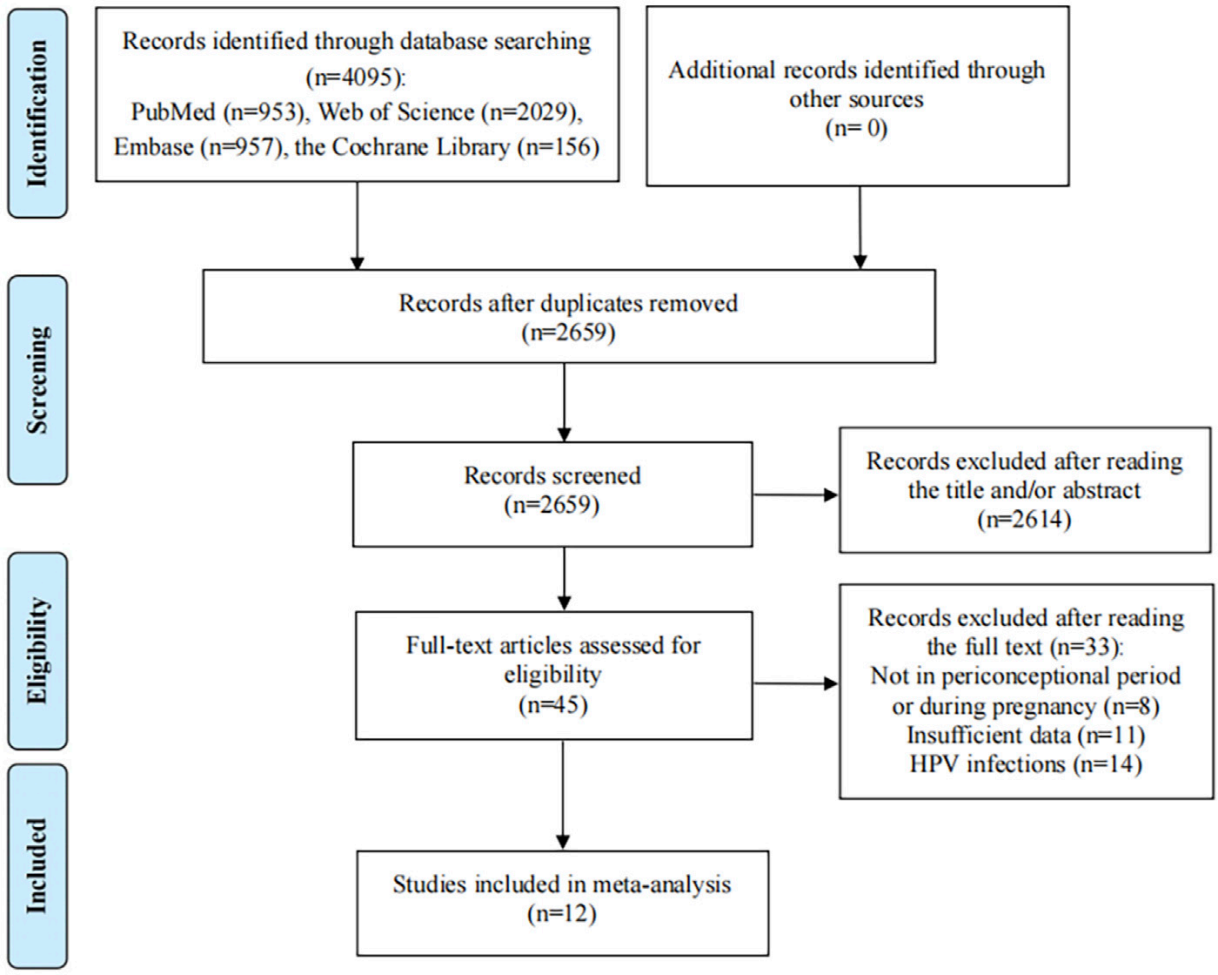
Flow diagram of the process of selection of articles.

Depending on the search strategy, 4,095 studies were identified. After eliminating the duplicates, 2,659 records remained. Of these, 2,614 studies excluded for their titles or abstracts being not relevant, and 45 full texts were assessed for eligibility. After reading the full text, 33 articles did not meet the inclusion criteria: 11 studies provided insufficient outcome data, 8 articles reported HPV vaccination not in periconceptional period or during pregnancy; 14 articles reported the pregnancy outcomes of HPV infections rather than HPV vaccination. Finally, 12 eligible studies were included in the present meta-analysis (Figure 1) (Garland et al., 2009; Kharbanda et al*.*, 2021; Angelo et al., 2014; Baril et al., 2015; Panagiotou et al., 2015; Scheller et al., 2017; Kharbanda et al., 2018; Lipkind et al*.*, 2017; Moreira et al*.*, 2016; Chen et al., 2019; Faber et al., 2019; Bukowinski et al., 2020).

### Characteristics and quality assessment of the included studies

The main characteristics of included researches and research participants were summarized in Table 1. All of included studies were RCTs and cohort studies. Three articles reported pooled results, including pooled analysis of 42 (conducted in 40 countries), seven (conducted in 31 countries), and five trials (conducted in multiple countries). There were three studies, eight studies and two studies, focused on the effect of 2vHPV, 4vHPV, and 9vHPV vaccine, respectively. Eligible participants were women who received HPV vaccine in periconceptional period or during pregnancy. Two RCTs were considered as low quality. Eight cohort studies were assessed as high quality, because the study design had been described in detail (Supplementary Material S2).

**TABLE 1:** Characteristics of the included studies.

First author (Year)	Study design	Country/Region	Study time	Exposure group			Control group			Age	Duration of follow-up	Outcomes
				N	Vaccination exposure time	Type of vaccine	N	Vaccination exposure time	Type of vaccine			
Kharbanda et al. (2018)	RCS	United States	2008.1–2014.11	1881	① peripregnancy:42 days before LMP; ② during pregnancy: first 19 weeks of pregnancy; ③ peri or during pregnancy: 42 days before LMP to 19 weeks of gestation	4vHPV vaccine	919	Vaccinated within 16–22 weeks before LMP	4vHPV vaccine	12–27 years	NR	1
Scheller et al. (2017)	RCS	Denmark	2006.10–2013.11	6,171	7–22 weeks of pregnancy	4vHPV vaccine	24,684	Not vaccinated during 7–22 weeks of pregnancy	4vHPV vaccine	NR	NR	1, 2, 3, 4, 5
Angelo et al. (2014)	Pooled analysis of RCTs	40 countries	The data lock point was 2011.4	982	① 45 days before and 30 days after pregnancy; ② 60 days before pregnancy to pregnancy end; ③ first 12 weeks of pregnancy	2vHPV vaccine	863	Vaccination with control vaccine within 60 days before pregnancy to pregnancy end	Control vaccines or placebo	15–25 years	0–9.4 years	1, 2, 3, 5, 6
Moreira et al. (2016)	Pooled analysis of RCTs	31 countries	NR	172	30 days before and after conception	9vHPV vaccine and 4vHPV vaccine	2,722	Not vaccinated during 30 days before and after conception	9vHPV vaccine and 4vHPV vaccine	16–26 years	NR	1, 2, 3, 6
Chen et al. (2019)	RCT	China	2009.1–2016.9	1,503	Gestation after HPV vaccination	4vHPV vaccine	1,503	Gestation after received placebo	Placebo	20–45 years	90 months	1, 2, 3, 6
Garland et al. (2009)	Pooled analysis of RCTs	Multiple countries	NR	2008	30 days before conception	4vHPV vaccine	2029	Vaccination with placebo within 30 days before conception	Placebo	16–45 years	0.6–3.7 years	1, 2, 3, 4, 5, 6
Panagiotou et al. (2015)	PCS	Costa Rica	2004.6–2013.12	381	90 days before and after conception	2vHPV vaccine	3,227	Not vaccinated HPV vaccine	Hepatitis A vaccine or unvaccination	18–25 years	4 years	1
Lipkind et al. (2017)	RCS	United States	2007.1–2013.9	1,358	① 2 weeks before to 2 weeks after LMP; ② 2–28 weeks of gestation	4vHPV vaccine	8,196	Vaccinated 4–18 months before LMP	4vHPV vaccine	13–27 years	NR	3, 4, 5
Baril et al. (2015)	RCS	United Kingdom	2008.9–2011.6	330	90 days before and 30 days after LMP	2vHPV vaccine	632	Vaccinated within 4–18 months before LMP	2vHPV vaccine	15–25 years	NR	1, 2, 3, 4, 5
Faber et al. (2019)	RCS	Denmark	2006.10–2014.12	7,487	① 4 weeks before conception to 22 weeks of gestation; ② 4 weeks before conception to birth	4vHPV vaccine	479,298	Not vaccinated from 4 weeks before conception to birth	4vHPV vaccine	NR	NR	1, 2
Bukowinski et al. (2020)	RCS	United States	2007–2014	1775	Received 4vHPV on or after estimated date of LMP through the end of pregnancy	4vHPV vaccine	8,008	Vaccinated 4–12 months prior to date of LMP	4vHPV vaccine	17–28 years	NR	1, 3, 5
Kharbanda et al. (2021)	RCS	United States	2015.10–2018.11	941	① From 42 days before LMP until LMP; ② from LMP to 19 completed weeks’ gestation	9vHPV vaccine	552	Vaccinated from 22 to 16 weeks before LMP	9vHPV vaccine or 4vHPV vaccine	12–28 years	Follow-up until 1 year after pregnancy end	1, 3, 4, 5

N, number; RCS, retrospective cohort study; PCS, prospective cohort study; LMP, last menstrual period; 2vHPV, bivalent human papillomavirus; 4vHPV, quadrivalent human papillomavirus; 9vHPV, 9-valent human papillomavirus; NR, not reported; RCT, randomized controlled trial; 1, spontaneous abortion; 2, stillbirth; 3, birth defects; 4, small size for gestational age; 5, preterm birth; 6, ectopic pregnancy.

### Pooled effect of adverse pregnancy outcomes in RCTs

**FIGURE 2:**
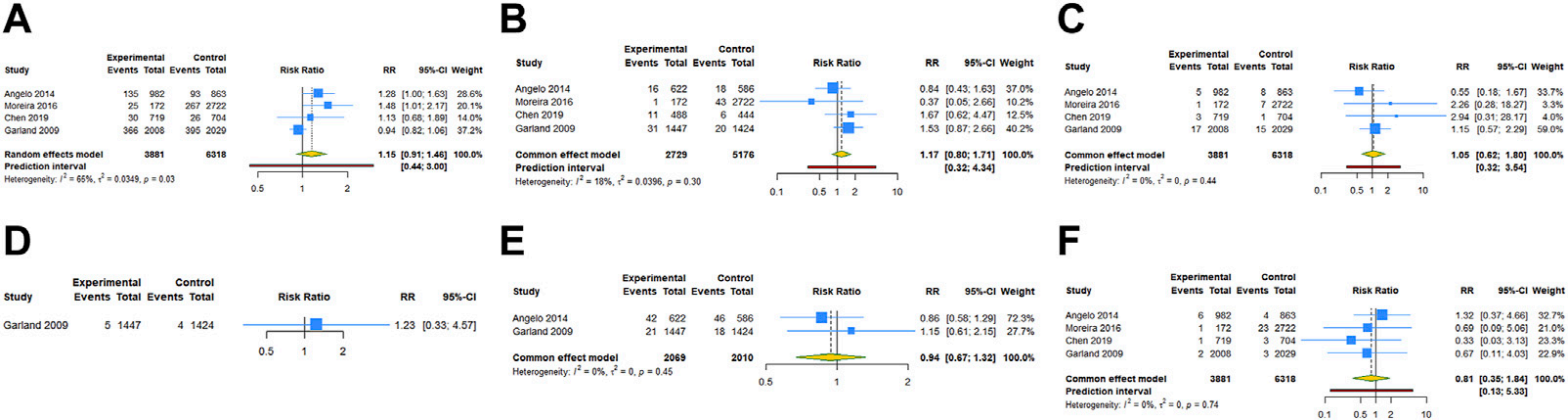
Forest plot of adverse pregnancy outcomes in RCTs. **(A)** Spontaneous abortion. **(B)** Birth defects. **(C)** Stillbirth. **(D)** Small size for gestational age. **(E)** Preterm birth. **(F)** Ectopic pregnancy.

Four RCTs examined the association between HPV vaccination and spontaneous abortion. The random-effects pooled estimate showed no significant association between HPV vaccination and spontaneous abortion (RR = 1.152, 95% CI: 0.909–1.460, 95% PI: 0.442–3.000), with significant heterogeneity (I^2^ = 65.2%, Tau^2^ = 0.0349) (Table 2; Figure 2A). Four RCTs compared birth defects between periconceptional or pregnancy women vaccinated HPV vaccine and those who were not. The difference in birth defects following HPV vaccination was not statistically significant (RR = 1.171, 95% CI: 0.802–1.709; 95% PI: 0.320–4.342, I^2^ = 17.9%, Tau^2^ = 0.0396) (Table 2; Figure 2B).

**TABLE 2:** Pooled effect of adverse pregnancy outcomes in randomized controlled trials and cohort studies.

Study design and outcomes	Number of study	Women (exposure/Control)	Meta-analysis				Heterogeneity	
			RR	95% CI	*p*-Value	95% PI	I^2^, Tau^2^	*p*-Value
Randomized controlled trial								
Spontaneous abortion	4	3,881/6,318	1.152	0.909–1.460	0.241	0.442–3.000	65.2%, 0.0349	0.035
Birth defects	4	2,729/5,176	1.171	0.802–1.709	0.415	0.320–4.342	17.9%, 0.0396	0.301
Stillbirth	4	3,881/6,318	1.053	0.616–1.800	0.851	0.318–3.540	0%, 0	0.445
Small size for gestational age	1	1,447/1,424	1.230	0.331–4.572	0.757			
Preterm birth	2	2069/2010	0.940	0.670–1.318	0.720	—	0%, 0	0.447
Ectopic pregnancy	4	3,881/6,318	0.807	0.353–1.842	0.610	0.128–5.335	0%, 0	0.739
Cohort study								
Spontaneous abortion	7	52,503/3280744	0.987	0.854–1.140	0.856	0.652–1.493	68.5%, 0.0205	0.004
Birth defects	5	5,402/22,346	0.960	0.697–1.322	0.801	0.371–2.480	52.2%, 0.0623	0.079
Stillbirth	3	5,991/311,646	1.033	0.651–1.639	0.891	0.052–21.064	0%, 0	0.643
Small size for gestational age	4	4,185/16,325	0.971	0.873–1.081	0.591	0.657–1.462	20.8%, 0.0040	0.286
Preterm birth	5	6,129/24,450	0.977	0.874–1.092	0.680	0.651–1.444	36.0%, 0.0099	0.181

Stillbirth was evaluated in four studies. HPV vaccination in periconceptional period or during pregnancy was not associated with an elevated risk of stillbirth (RR = 1.053, 95% CI: 0.616–1.800, 95% PI: 0.318–3.540; I^2^ = 0, Tau^2^ = 0) (Table 2; Figure 2C). Only one study reported the association between HPV vaccination and small size for gestational age (SGA). In the study, the RR of HPV vaccination for SGA was 1.230 (95% CI = 0.331–4.572) (Table 2; Figure 2D). Two RCTs assessed preterm birth in HPV vaccine vaccinated/unvaccinated pregnancies. Compared with the unexposed pregnancies, HPV vaccination pregnancies were not associated with higher risk for preterm birth (RR = 0.940, 95% CI: 0.670–1.318; I^2^ = 0, Tau^2^ = 0) (Table 2; Figure 2E). Four studies reported the association between HPV vaccination and ectopic pregnancy. The results showed HPV vaccination in periconceptional period or during pregnancy seem to decrease the risk of ectopic pregnancy, but without statistical significance (RR = 0.807, 95% CI: 0.353–1.842, 95% PI: 0.128–5.335; I^2^ = 0, and Tau^2^ = 0) (Table 2; Figure 2F).

### Pooled effect of adverse pregnancy outcomes in cohort studies

**FIGURE 3:**
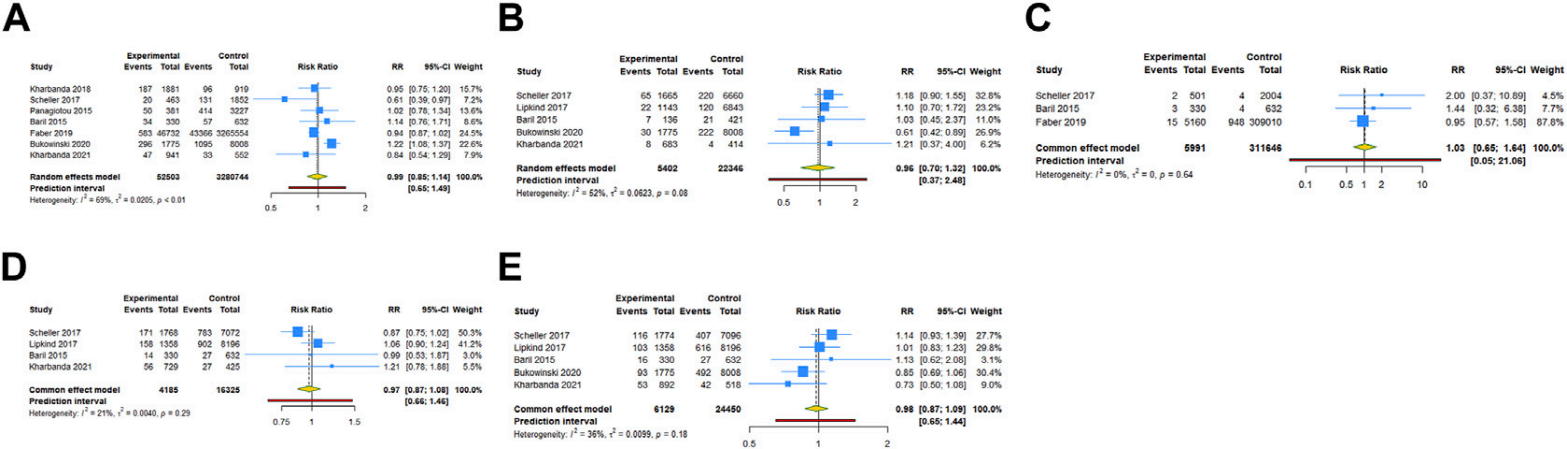
Forest plot of adverse pregnancy outcomes in cohort studies. **(A)** Spontaneous abortion. **(B)** Birth defects. **(C)** Stillbirth. **(D)** Small size for gestational age. **(E)** Preterm birth.

Seven cohort studies examined the association between HPV vaccination and spontaneous abortion. The results with a random-effect model showed that HPV vaccination in periconceptional period or during pregnancy seem to reduce the risk of spontaneous abortion, but without statistical significance (RR = 0.987, 95% CI: 0.854–1.140, 95% PI: 0.652–1.493; I^2^ = 68.5%, Tau^2^ = 0.0205) (Table 2; Figure 3A). Five studies reported the association between HPV vaccine exposure and birth defects. The result suggested that HPV vaccination in periconceptional period or during pregnancy did not increase the risk of birth defects with pooled RR of 0.960 (95% CI: 0.697–1.322, 95% PI: 0.371–2.480; I^2^ = 52.2%, Tau^2^ = 0.0623) (Table 2; Figure 3B).

Three cohort studies assessed stillbirth in HPV vaccine exposed/unexposed pregnancies. The pooled RR was 1.033 (95% CI: 0.651–1.639, 95% PI: 0.052–21.064; I^2^ = 0, Tau^2^ = 0), indicating HPV vaccine exposed pregnancies were associated with no higher risk for stillbirth (Table 2; Figure 3C). Four studies reported the association between HPV vaccination and SGA. The result showed HPV vaccination in periconceptional period or during pregnancy seem to decrease the risk of SGA, but without statistical significance (RR = 0.971, 95% CI: 0.873–1.081, 95% PI: 0.657–1.462; I^2^ = 20.8%, Tau^2^ = 0.0040) (Table 2; Figure 3D). Five cohort studies compared preterm birth between periconceptional or pregnancy women vaccinated HPV vaccine and those who were not. Among women with HPV vaccination as opposed to those who were not vaccinated, a nonsignificant decrease in preterm birth was demonstrated (RR = 0.977, 95% CI: 0.874–1.092, 95% PI: 0.651–1.444; I^2^ = 36.0%, Tau^2^ = 0.0099) (Table 2; Figure 3E).

## Subgroup analysis of adverse pregnancy outcomes in RCTs and cohort studies

**FIGURE 4:**
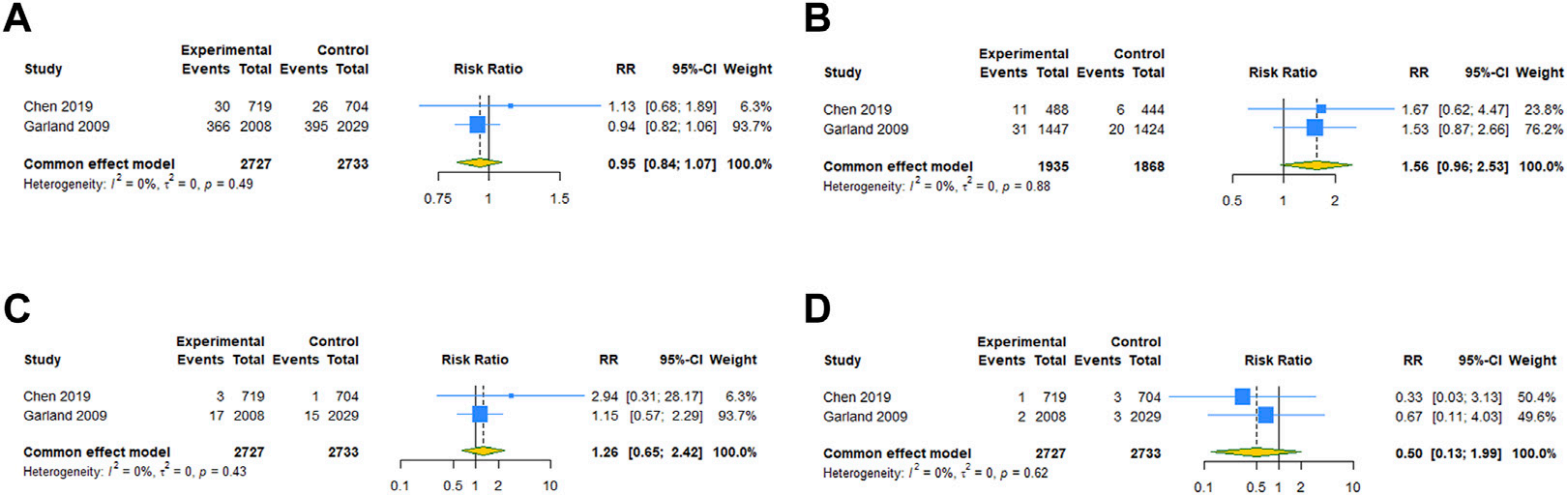
Subgroup analysis of adverse pregnancy outcomes after 4vHPV vaccination in RCTs. **(A)** Spontaneous abortion. **(B)** Birth defects. **(C)** Stillbirth. **(D)** Ectopic pregnancy.

**FIGURE 5:**
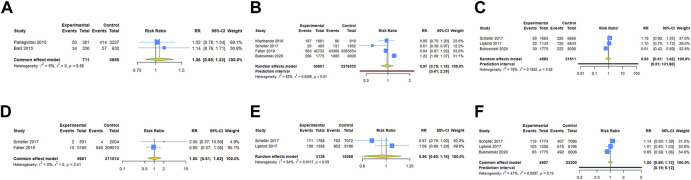
Subgroup analysis of adverse pregnancy outcomes after 2vHPV or 4vHPV vaccination in cohort studies. **(A)** Spontaneous abortion after 2vHPV vaccination. **(B)** Spontaneous abortion, **(C)** birth defects, **(D)** stillbirth, **(E)** small size for gestational age, and **(F)** preterm birth after 4vHPV vaccination.

For the subgroups with ≥2 studies included, we conducted a subgroup analysis by the type of vaccine. The results of RCTs showed that 4vHPV vaccination in periconceptional period or during pregnancy did not increase the risk of spontaneous abortion (RR = 0.948, 95% CI: 0.838–1.074; I^2^ = 0, Tau^2^ = 0), birth defects (RR = 1.559, 95% CI: 0.960–2.533; I^2^ = 0, Tau^2^ = 0), stillbirth (RR = 1.259, 95% CI: 0.654–2.425; I^2^ = 0, Tau^2^ = 0), and ectopic pregnancy (RR = 0.499, 95% CI: 0.125–1.988; I^2^ = 0, Tau^2^ = 0) (Table 3; Figures 4A–D). Subgroup analysis of cohort studies suggested that 2vHPV (RR = 1.060, 95% CI: 0.845–1.329; I^2^ = 0, Tau^2^ = 0) or 4vHPV vaccine (RR = 0.970, 95% CI: 0.794–1.184, 95% PI: 0.410–2.292; I^2^ = 83.3%, Tau^2^ = 0.0296) exposure was not associated with the increased risk of spontaneous abortion (Table 3; Figures 5A, B). In cohort studies, 4vHPV vaccination in periconceptional period or during pregnancy did not increase the risk of birth defects (RR = 0.930, 95% CI: 0.609–1.421, 95% PI: 0.007–131.804; I^2^ = 75.7%, Tau^2^ = 0.1052), stillbirth (RR = 0.999, 95% CI: 0.614–1.624; I^2^ = 0, Tau^2^ = 0), SGA (RR = 0.961, 95% CI: 0.797–1.158; I^2^ = 64.3%, Tau^2^ = 0.0117), and preterm birth (RR = 0.996, 95% CI: 0.885–1.121, 95% PI: 0.195–5.116; I^2^ = 46.9%, Tau^2^ = 0.0097) (Table 3; Figures 5C–F).

**TABLE 3:** Subgroup analysis of adverse pregnancy outcomes in randomized controlled trials and cohort studies.

Study design and outcomes	Number of study	Women (exposure/Control)	Meta-analysis				Heterogeneity	
			RR	95% CI	*p*-Value	95% PI	I^2^, Tau^2^	*p*-Value
Randomized controlled trial								
4vHPV vaccine								
Spontaneous abortion	2	2,727/2,733	0.948	0.838–1.074	0.404	—	0%, 0	0.487
Birth defects	2	1935/1868	1.559	0.960–2.533	0.073	—	0%, 0	0.877
Stillbirth	2	2,727/2,733	1.259	0.654–2.425	0.491	—	0%, 0	0.434
Ectopic pregnancy	2	2,727/2,733	0.499	0.125–1.988	0.324	—	0%, 0	0.622
Cohort study								
2vHPV vaccine								
Spontaneous abortion	2	711/3,859	1.060	0.845–1.329	0.615	—	0%, 0	0.657
4vHPV vaccine								
Spontaneous abortion	4	50,851/3276333	0.970	0.794–1.184	0.762	0.410–2.292	83.3%, 0.0296	<0.001
Birth defects	3	4,583/21,511	0.930	0.609–1.421	0.739	0.007–131.804	75.7%, 0.1052	0.016
Stillbirth	2	5,661/311,014	0.999	0.614–1.624	0.997	—	0%, 0	0.408
Small size for gestational age	2	3,126/15,268	0.961	0.797–1.158	0.674	—	64.3%, 0.0117	0.094
Preterm birth	3	4,907/23,300	0.996	0.885–1.121	0.952	0.195–5.116	46.9%, 0.0097	0.1521

### Trial sequential analysis results

**FIGURE 6:**
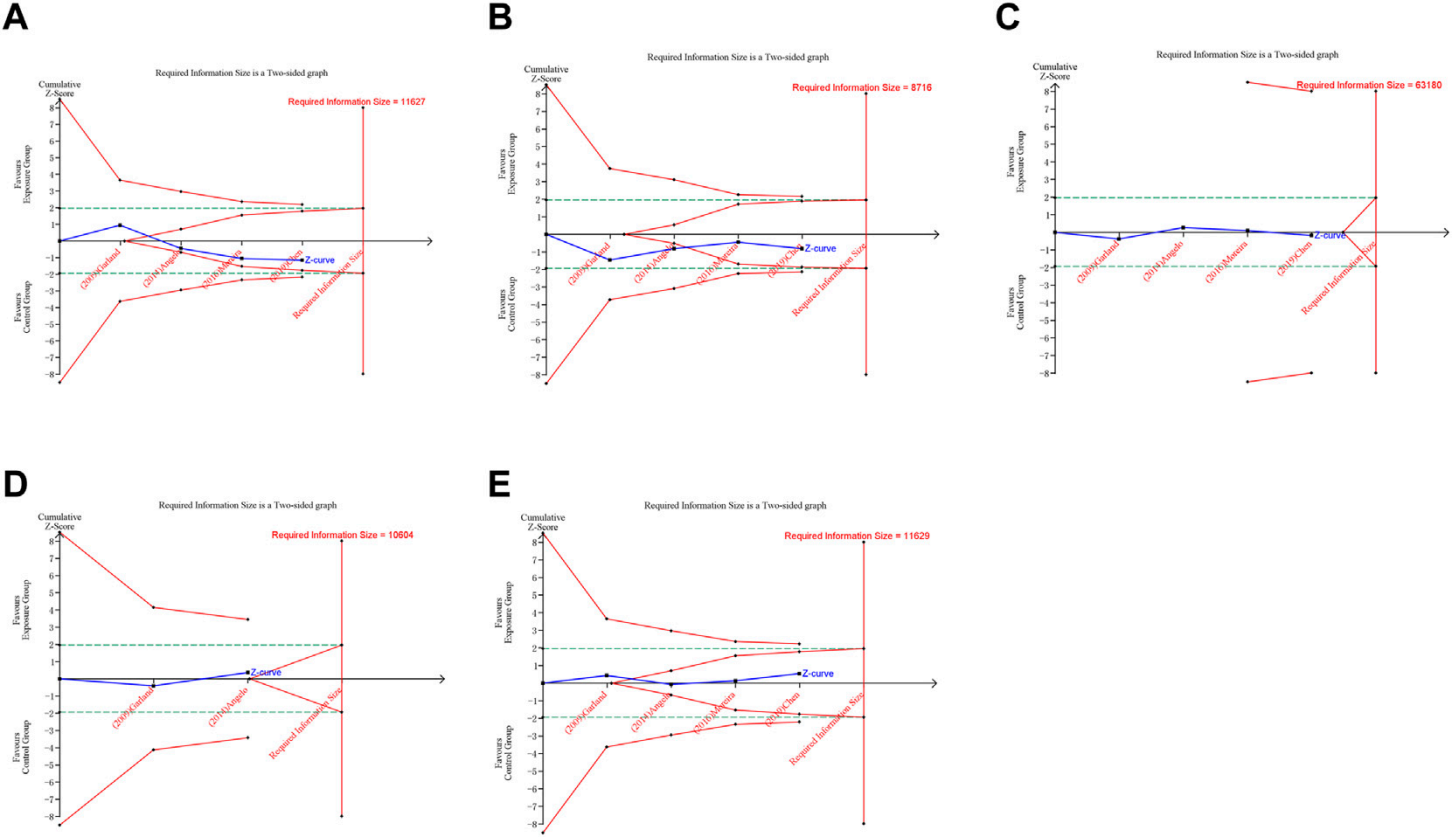
Trial sequential analysis (TSA) of adverse pregnancy outcomes in RCTs. **(A)** Spontaneous abortion. **(B)** Birth defects. **(C)** Stillbirth. **(D)** Preterm birth. **(E)** Ectopic pregnancy. Uppermost and lowermost red curves represent trial sequential monitoring boundary lines for benefit and harm, respectively. Horizontal green lines represent the conventional boundaries for statistical significance. Inner red lines represent the futility boundary.

**FIGURE 7:**
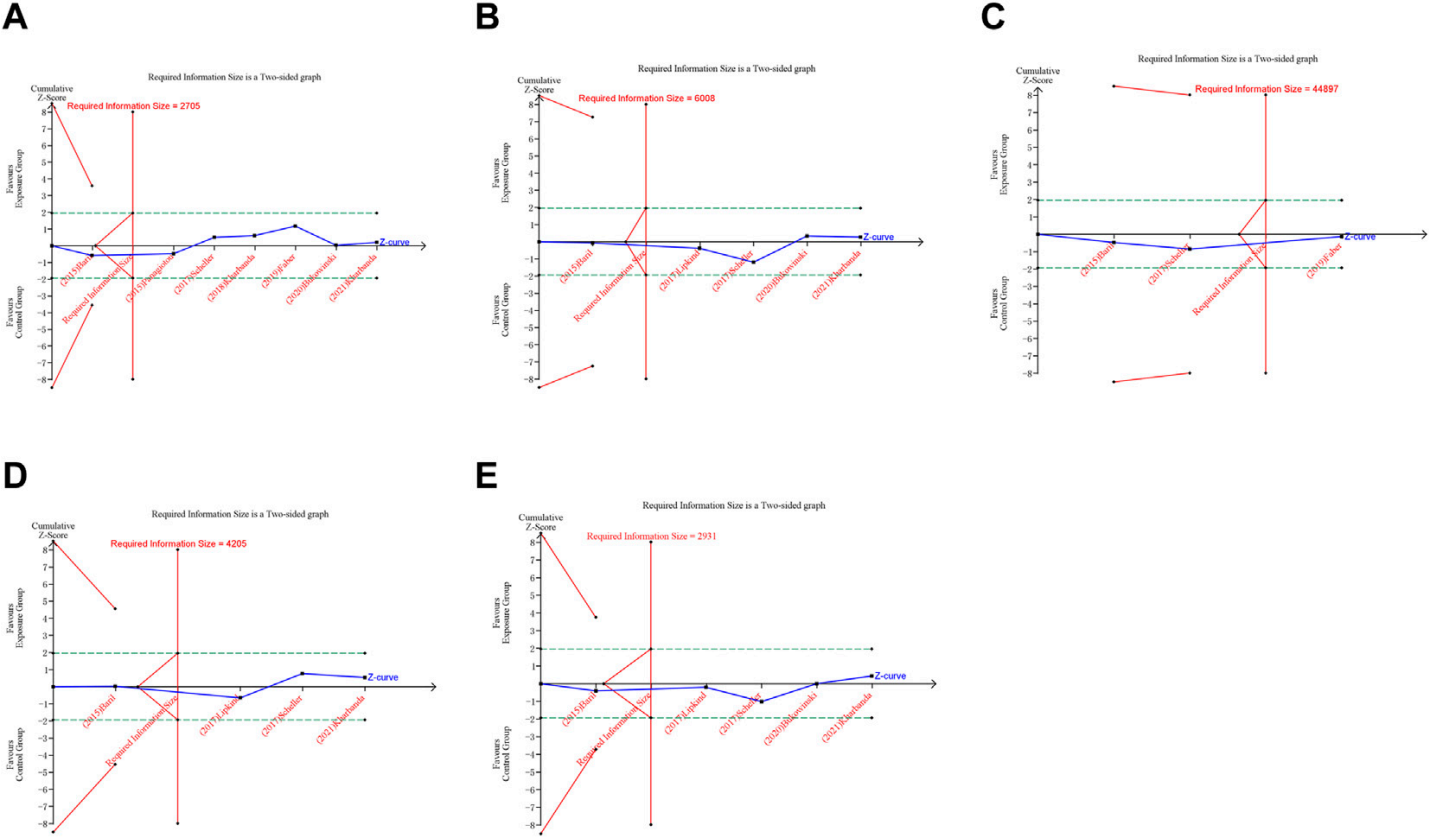
Trial sequential analysis (TSA) of adverse pregnancy outcomes in cohort studies. **(A)** Spontaneous abortion. **(B)** Birth defects. **(C)** Stillbirth. **(D)** Small size for gestational age. **(E)** Preterm birth. Uppermost and lowermost red curves represent trial sequential monitoring boundary lines for benefit and harm, respectively. Horizontal green lines represent the conventional boundaries for statistical significance. Inner red lines represent the futility boundary.

In trial sequential analysis of RCTs, we observed that all the cumulative Z-curves did not cross the trial sequential monitoring boundary and RIS boundary, suggesting that we cannot draw a definitive conclusion about spontaneous abortion, birth defects, stillbirth, preterm birth and ectopic pregnancy in RCTs due to the presence of false positive (Figure 6). For cohort studies, the cumulative Z-curve significantly crossed the RIS boundary, but did not cross the trial sequential monitoring boundary, suggesting that a relatively definite conclusion of spontaneous abortion, birth defects, stillbirth, SGA and preterm birth can be obtained in cohort studies (Figure 7).

### Publication bias and sensitivity analysis

We conducted publication bias test and sensitivity analysis for the pooled result of spontaneous abortion which included seven studies. Begg’s test and Egger’s test were performed to evaluate the publication bias and the results indicated that no significant publication bias existed in cohort studies (Begg’s test: *p* = 0.764, Egger’s test: *p* = 0.784). The funnel plot was shown in Supplementary Figure S1 (Supplementary Material S3). Sensitivity analysis was performed by calculating the pooled RRs and the corresponding 95% CIs after individual studies were omitted to assess whether the pooled results were affected by a single study. The sensitivity analysis indicated that Bukowinski’s study may be the cause of high heterogeneity (Supplementary Figure S1, Supplemental Material S3).

## Discussion

The number of women in the world who were inadvertently vaccinated with HPV in periconceptional period or during pregnancy was enormous. If HPV vaccination during periconceptional period or during pregnancy increases the risk of adverse pregnancy outcomes, even if the risk is very small, we should be vigilant. Although several meta-analyses have reported the relationship between HPV vaccination in the periconceptional period or during pregnancy and adverse pregnancy outcomes (Tan et al*.*, 2019; Wang et al., 2020), our meta-analysis included four recent studies to update previous results, and further performed meta-analysis of RCT and retrospective cohort studies, respectively. The results indicated that HPV vaccination in periconceptional period or during pregnancy did not increase the risks of adverse pregnancy outcomes, including spontaneous abortion, birth defects, stillbirth, SGA, preterm birth and ectopic pregnancy.

Previous studies on the safety of HPV exposure around conception or during pregnancy included combined analyses of clinical trials, large observational cohort studies and post-marketing surveillance pregnancy registries. Among these various research methods and populations, there were no indications of increased risks for spontaneous abortion (Goss et al., 2015), birth defects (Moro et al., 2015), preterm birth (Forinash et al., 2011) or SGA (Scheller et al*.*, 2017) after exposure to HPV vaccines during pregnancy. However, the majority of prior studies have only reported the relationship between exposure of 4vHPV vaccine during pregnancy and adverse pregnancy outcomes, the evidence of the association between 2vHPV vaccination and adverse pregnancy outcomes was limited. There were only one RCT and two cohort studies, focused on the effect of 2vHPV in our analysis. No association was found between 2vHPV exposure around conception and spontaneous abortion. There is no established pathophysiological mechanism by which 2vHPV vaccination would affect the risk of spontaneous abortion (Panagiotou et al*.*, 2015). A theoretical debate involves alterations in the maternal immune system during early gestation caused by ASO4 adjuvant, which is composed of aluminum phosphate and monophosphoryl lipid A (Goldhaber and Fireman, 1991). Nevertheless, other ASO4 based vaccines were not associated with the risk of spontaneous abortion (Tavares et al., 2013). More generally, the evidence of a causal effect of autoimmunity itself on the risk of spontaneous abortion is weak (Larsen et al., 2013). Also, the vaccine was not associated with autoimmune conditions related to abortion (e.g., antiphospholipid syndrome and thyroid autoimmunity) (Panagiotou et al*.*, 2015).

Policymakers have published reassuring reports on the safety of 2vHPV and 4vHPV vaccines, both overall and for pregnancy related outcomes specifically. A review of the latest evidence used in the recommendations of the Advisory Committee on Immunization Practice concluded that the public health benefits of HPV vaccination outweigh the potential harms (Oshman and Davis, 2020). Our pooled analysis suggested that 4vHPV vaccination in periconceptional period or during pregnancy was not associated with the increased risks of spontaneous abortion, birth defects, stillbirth, ectopic pregnancy, SGA and preterm birth. A previous analysis of pregnancy outcomes in women with 4vHPV vaccination in a global clinical program found similar incidence of adverse pregnancy outcomes in the 4vHPV vaccine and placebo groups without evidence of a negative effect of vaccination on pregnancy outcomes (Garland et al*.*, 2009). Post-marketing registry data indicated that 4vHPV vaccine exposure around conception or during pregnancy was not associated with an increased risk of adverse pregnancy outcomes, such as spontaneous abortion or birth defects (Goss et al*.*, 2015; Sy et al., 2018). Given the large amount of evidence that suggests accidental HPV vaccination during pregnancy, including 4vHPV, does not cause a risk to the pregnancy or developing fetus, it was suggested that HPV vaccination be included in routine prenatal care, as this is a time when women regularly encounter the healthcare system (Berenson et al., 2014). Although HPV vaccination before initiation of sexual activity is most effective, study has showed that HPV vaccination can provide protection against HPV-related dysplasia even among women who have previously been exposed to and/or infected with HPV (Bukowinski et al*.*, 2020).

2vHPV, 4vHPV and 9vHPV vaccines are all recombinant, contain virus-like particles, and are enhanced by adjuvants that trigger higher immune responses than natural infections (Bonde et al*.*, 2016). Despite these HPV vaccines are noninfectious recombinant vaccines, excipients need to be considered in addition to the types of recombinant HPV when determining maternal and fetal safety (Forinash et al*.*, 2011). Both 4vHPV and 9vHPV vaccines contain an amorphous aluminum hydroxyphosphate sulfate adjuvant which is contained in other products made by Merck, such as *Haemophilus* influenzae B conjugate vaccine, hepatitis A vaccine and hepatitis B vaccine. These products are appropriate for use in pregnancy (Forinash et al*.*, 2011). Given this fact, 4vHPV and 9vHPV vaccines appear to be relatively safe from the excipient standpoint. Due to the limited number of included studies, the pooled results of the association between 9vHPV vaccination and adverse pregnancy outcomes both in RCTs and cohort studies cannot be obtained. 9vHPV vaccine was generally well tolerated in clinical trials, with adverse event profile similar to that of 4vHPV vaccine. Discontinuations due to adverse events and serious vaccine-related adverse events were rare (Moreira et al*.*, 2016). We believe that this meta-analysis supports the current recommendations of the Advisory Committee on Immunization Practices that although 9vHPV vaccine is not recommended to be used during pregnancy, it can be administered to women of childbearing age without routine pregnancy testing (Kharbanda et al., 2021).

Although there has been ample evidence of vaccine safety from post-marketing surveillance studies and clinical trials, public misperceptions and concerns about the safety of vaccines may hinder the implementation of HPV vaccination programs (Chen et al*.*, 2019). Immunization-related anxiety reactions have occurred in some regions, adversely affecting HPV vaccination programs and leaving young female individuals vulnerable to preventable HPV-related diseases (Hanley et al., 2015; Tanaka et al., 2016). However, concerns regarding inadvertent HPV vaccination in periconceptional period or during pregnancy may further decrease, given the increasing intensity of HPV vaccination at the recommended age of 11–12 years (Cullen et al., 2014). Preparing, facilitating communication and enhancing vaccine infrastructure can ensure the implementation of high coverage and sustainable vaccination schedules (Chen et al*.*, 2019).

The present study leaded to some meaningful implications, it yet has some limitations. Firstly, some of these studies included pregnancies with small sample sizes, and there were no or limited adjustments for factors affecting pregnancy outcomes and malformations, which may bias the conclusions. Secondly, the majority of the studies included in present meta-analysis did not clearly divide fetal development stages at which HPV vaccine administered or doses of HPV vaccination. This information should be reported and analyzed to further evaluate the safety of HPV vaccine exposure around conception or during pregnancy. Thirdly, the safety profiles from included studies were based primarily on 2vHPV or 4vHPV vaccination, and very few studies evaluated the 9vHPV vaccine exposures in periconceptional period or during pregnancy. Fourthly, most of the included studies reported the association between HPV vaccination in periconceptional period (including before and after conception) and adverse pregnancy outcomes, and it was unclear whether HPV vaccines was administered before or after conception in the majority of studies. Only two RCTs explicitly reported the association between HPV vaccination before conception and adverse pregnancy outcomes, and the results showed that HPV vaccination before conception did not increase the risks of spontaneous abortion, birth defects, stillbirth and ectopic pregnancy. More studies are need to be included for further subgroup analysis by vaccination exposure time.

## Conclusion

This meta-analysis demonstrated that HPV vaccine exposures in periconceptional period or during pregnancy did not increase the risks of adverse pregnancy outcomes, such as spontaneous abortion, birth defects, stillbirth, SGA, preterm birth and ectopic pregnancy. Moreover, periconceptional or pregnancy exposure of 2vHPV vaccine was not associated with the increased risk of spontaneous abortion, and 4vHPV vaccination around conception or during pregnancy was not associated with the increased risk of spontaneous abortion, birth defects, stillbirth, SGA, preterm birth and ectopic pregnancy. Despite the limited studies included in present analysis, our meta-analysis revealed no association between 9vHPV vaccination in periconceptional period or during pregnancy and spontaneous abortion, birth defects, SGA and preterm birth.

## Data Availability

The original contributions presented in the study are included in the article/Supplementary Material. Further inquiries can be directed to the corresponding authors.
