# Supplementary material for: Association of periconceptional or pregnancy exposure of HPV vaccination and adverse pregnancy outcomes: a systematic review and meta-analysis with trial sequential analysis
Source: Front Pharmacol. 2023 May 9;14:1181919. doi: 10.3389/fphar.2023.1181919 (PMC10203546; doi:10.3389/fphar.2023.1181919)
Supplement: Supplementary file 1 [file Table1.DOCX]

**Pubmed: 953 citations found (Search date: January 30, 2023. Updated date: March 5, 2023)**

#1 Pregnant Women[mh] OR Pregnancy[mh] OR Pregnan*[tiab] OR conception[tiab] OR gravid*[tiab] OR gestation*[tiab] OR Childbearing[tiab] OR Child bearing[tiab] OR parturient[tiab] OR parturition[tiab]

#2 Papilloma[mh] OR Papillomaviridae[mh:noexp] OR Alphapapillomavirus[mh] OR Papilloma*[tiab] OR Alphapapillomavirus*[tiab] OR HPV[tiab]

#3 Infant, Premature[mh] OR Infant, Low Birth Weight[mh] OR preterm[tiab] OR premature[tiab] OR prematurity[tiab] OR low birthweight[tiab] OR low-birth weight[tiab] OR LBW[tiab] OR VLBW[tiab] OR ELBW[tiab] OR small for gestational age[tiab] OR Small for Gestation Age[tiab] OR smallness for gestational age[tiab] OR SGA[tiab] OR hypertensive disorder*[tiab] OR Hypertension[tiab] OR preterm[OT] OR premature[OT] OR prematurity[OT] OR low birthweight[OT] OR low-birth weight[OT] OR LBW[OT] OR VLBW[OT] OR ELBW[OT] OR small for gestational age[OT] OR Small for Gestation Age[OT] OR "smallness for gestational age"[OT] OR SGA[OT] OR hypertensive disorder*[OT] OR Hypertension[OT] OR Pregnancy Outcome[mh] OR Pregnancy Complications[mh] OR pregnancy outcome*[tiab] OR pregnancy complication*[tiab] OR pregnancy adverse outcome*[tiab] OR pregnancy loss*[tiab] OR pregnancy toxemia*[tiab] OR pregnancy toxaemia*[tiab] OR Gestational hypertension[tiab] OR Abortion*[tiab] OR Miscarriage*[tiab] OR Stillbirth*[tiab] OR Still-birth*[tiab] OR Perinatal outcome*[tiab] OR Perinatal complication*[tiab] OR Perinatal adverse outcome*[tiab] OR Obstetric outcome*[tiab] OR Obstetric complication*[tiab] OR Obstetric adverse outcome*[tiab] OR Preeclampsia*[tiab] OR Eclampsia*[tiab] OR Maternal outcome*[tiab] OR Maternal complication*[tiab] OR Maternal adverse outcome*[tiab] OR "Reproductive outcome"[tiab] OR "Reproductive outcomes"[tiab] OR HELLP syndrome[tiab] OR fetal growth restriction[tiab] OR fetal growth retardation[tiab] OR foetal growth restriction[tiab] OR foetal growth retardation[tiab] OR premature rupture of membrane*[tiab] OR "premature rupture of fetal membrane"[tiab] OR pregnancy outcome*[OT] OR pregnancy complication*[OT] OR pregnancy adverse outcome*[OT] OR pregnancy loss*[OT] OR pregnancy toxemia*[OT] OR pregnancy toxaemia*[OT] OR Gestational hypertension[OT] OR Abortion*[OT] OR Miscarriage*[OT] OR Stillbirth*[OT] OR Still-birth*[OT] OR Perinatal outcome*[OT] OR Perinatal complication*[OT] OR Obstetric outcome*[OT] OR Obstetric complication*[OT] OR Obstetric adverse outcome*[OT] OR "Reproductive outcome"[OT] OR "Reproductive outcomes"[OT] OR Preeclampsia*[OT] OR Eclampsia*[OT] OR Maternal outcome*[OT] OR Maternal complication*[OT] OR HELLP syndrome[OT] OR fetal growth restriction[OT] OR fetal growth retardation[OT] OR foetal growth restriction[OT] OR "foetal growth retardation"[OT] OR premature rupture of membrane*[OT] OR "premature rupture of fetal membrane"[OT]

#4 (#1 AND #2 AND #3)

**Web of Science: 2029 citations found (Search date: January 30, 2023. Updated date: March 5, 2023)**

#1 TS=(Papilloma* OR Alphapapillomavir* OR HPV OR Genital Wart* OR Venereal Wart* OR Anogenital Wart* OR Ano-genital Wart* OR Anal Wart* OR Condylomata Acuminat*)

#2 TS=(pregnancy outcome* OR pregnancy complication* OR pregnancy adverse outcome* OR pregnancy loss* OR pregnancy toxemia* OR pregnancy toxaemia* OR Gestational hypertension OR Abortion* OR Miscarriage* OR Stillbirth* OR Still-birth* OR Perinatal outcome* OR Perinatal complication* OR Perinatal adverse outcome* OR Obstetric outcome* OR Obstetric complication* OR Obstetric adverse outcome* OR Preeclampsia* OR Eclampsia* OR Maternal outcome* OR Maternal complication* OR Maternal adverse outcome* OR Reproductive adverse outcome* OR Reproductive outcome* OR HELLP syndrome OR fetal growth restriction OR fetal growth retardation OR foetal growth restriction OR foetal growth retardation OR premature rupture of membrane* OR premature rupture of fetal membrane OR premature rupture of foetal membrane OR Poor Fetal Growth OR Poor Foetal Growth OR Infant, Premature OR Infant, Low Birth Weight OR preterm OR premature OR prematurity OR low birthweight OR low-birth weight OR LBW OR VLBW OR ELBW OR small for gestational age OR Small for Gestation Age OR smallness for gestational age OR SGA OR hypertensive disorder* OR Hypertension)

#3 TS=(Pregnan* OR conception OR gravid* OR gestation* OR Childbearing OR Child bearing OR parturient OR parturition OR expectant OR Placenta*)

#4 (#1 AND #2 AND #3)

**Embase: 957 citations found (Search date: January 30, 2023. Updated date: March 5, 2023)**

#1 papilloma*:ti,ab,kw OR alphapapillomavir*:ti,ab,kw OR hpv:ti,ab,kw OR 'genital wart*':ti,ab,kw OR 'venereal wart*':ti,ab,kw OR 'anogenital wart*':ti,ab,kw OR 'ano-genital wart*':ti,ab,kw OR 'anal wart*':ti,ab,kw OR 'condylomata acuminat*':ti,ab,kw OR 'papilloma'/exp OR 'papillomaviridae'/exp OR 'alphapapillomavirus'/exp OR 'papillomavirus infections'/exp

#2 pregnan*:ti,ab,kw OR conception:ti,ab,kw OR gravid*:ti,ab,kw OR gestation*:ti,ab,kw OR childbearing:ti,ab,kw OR 'child bearing':ti,ab,kw OR parturient:ti,ab,kw OR parturition:ti,ab,kw OR expectant:ti,ab,kw OR placenta*:ti,ab,kw OR 'pregnant women'/exp OR 'pregnancy'/exp

#3 'pregnancy outcome*':ti,ab,kw OR 'pregnancy complication*':ti,ab,kw OR 'pregnancy adverse outcome*':ti,ab,kw OR 'pregnancy loss*':ti,ab,kw OR 'pregnancy toxemia*':ti,ab,kw OR 'pregnancy toxaemia*':ti,ab,kw OR 'gestational hypertension':ti,ab,kw OR abortion*:ti,ab,kw OR miscarriage*:ti,ab,kw OR stillbirth*:ti,ab,kw OR 'still birth*':ti,ab,kw OR 'perinatal outcome*':ti,ab,kw OR 'perinatal complication*':ti,ab,kw OR 'perinatal adverse outcome*':ti,ab,kw OR 'obstetric outcome*':ti,ab,kw OR 'obstetric complication*':ti,ab,kw OR 'obstetric adverse outcome*':ti,ab,kw OR preeclampsia*:ti,ab,kw OR eclampsia*:ti,ab,kw OR 'maternal outcome*':ti,ab,kw OR 'maternal complication*':ti,ab,kw OR 'maternal adverse outcome*':ti,ab,kw OR 'reproductive adverse outcome*':ti,ab,kw OR 'reproductive outcome*':ti,ab,kw OR 'hellp syndrome':ti,ab,kw OR 'fetal growth restriction':ti,ab,kw OR 'fetal growth retardation':ti,ab,kw OR 'foetal growth restriction':ti,ab,kw OR 'foetal growth retardation':ti,ab,kw OR 'premature rupture of membrane*':ti,ab,kw OR 'premature rupture of fetal membrane':ti,ab,kw OR 'premature rupture of foetal membrane':ti,ab,kw OR 'poor fetal growth':ti,ab,kw OR 'poor foetal growth':ti,ab,kw OR 'pregnancy outcome'/exp OR 'pregnancy complications'/exp OR preterm:ti,ab,kw OR premature:ti,ab,kw OR prematurity:ti,ab,kw OR 'low birthweight':ti,ab,kw OR 'low-birth weight':ti,ab,kw OR lbw:ti,ab,kw OR vlbw:ti,ab,kw OR elbw:ti,ab,kw OR 'small for gestational age':ti,ab,kw OR 'small for gestation age':ti,ab,kw OR 'smallness for gestational age':ti,ab,kw OR sga:ti,ab,kw OR 'hypertensive disorder*':ti,ab,kw OR hypertension:ti,ab,kw OR 'infant, premature'/exp OR 'infant, low birth weight'/exp

#4 (#1 AND #2 AND #3)

**The Cochrane Library: 156 citations found (Search date: January 30, 2023. Updated date: March 5, 2023)**

#1 (Papilloma* OR Alphapapillomavir* OR HPV OR Genital Wart* OR Venereal Wart* OR Anogenital Wart* OR Ano-genital Wart* OR Anal Wart* OR Condylomata Acuminat*):ti,ab,kw OR (Papilloma OR papillomaviridae OR alphapapillomavirus OR wart virus OR papillomavirus infection)

#2 (Pregnancy Outcome OR Pregnancy Complication OR pregnancy disorder) OR (pregnancy outcome* OR pregnancy complication* OR pregnancy adverse outcome* OR pregnancy loss* OR pregnancy toxemia* OR pregnancy toxaemia* OR Gestational hypertension OR Abortion* OR Miscarriage* OR Stillbirth* OR Still-birth* OR Perinatal outcome* OR Perinatal complication* OR Perinatal adverse outcome* OR Obstetric outcome* OR Obstetric complication* OR Obstetric adverse outcome* OR Preeclampsia* OR Eclampsia* OR Maternal outcome* OR Maternal complication* OR Maternal adverse outcome* OR Reproductive adverse outcome* OR Reproductive outcome* OR HELLP syndrome OR fetal growth restriction OR fetal growth retardation OR foetal growth restriction OR foetal growth retardation OR premature rupture of membrane* OR "premature rupture of fetal membrane" OR "premature rupture of foetal membrane" OR Poor Fetal Growth OR Poor Foetal Growth):ti,ab,kw OR (Prematurity OR low birth weight) OR (preterm OR premature OR prematurity OR low birthweight OR low-birth weight OR LBW OR VLBW OR ELBW OR small for gestational age OR Small for Gestation Age OR smallness for gestational age OR SGA OR hypertensive disorder* OR Hypertension):ti,ab,kw (Word variations have been searched)

#3 (Pregnant Women OR Pregnancy) OR (Pregnan* OR conception OR gravid* OR gestation* OR Childbearing OR "Child bearing" OR parturient OR parturition OR expectant OR Placenta*):ti,ab,kw

#4 (#1 AND #2 AND #3)
