# Supplementary material for: Association of periconceptional or pregnancy exposure of HPV vaccination and adverse pregnancy outcomes: a systematic review and meta-analysis with trial sequential analysis
Source: Front Pharmacol. 2023 May 9;14:1181919. doi: 10.3389/fphar.2023.1181919 (PMC10203546; doi:10.3389/fphar.2023.1181919)
Supplement: Supplementary file 2 [file Table2.DOCX]

| TABLE S1 The Newcastle-Ottawa quality assessment scale of the included studies. | | | | | | | | | | | | |
| --- | --- | --- | --- | --- | --- | --- | --- | --- | --- | --- | --- | --- |
| Study | Selection | | | |  | Comparability | |  | Assessment of outcome | | | Total score |
|  | Representativeness of exposure arm(s) | Selection of the comparative arm(s) | Origin of exposure source | Demonstration that outcome of interest was not present at start of study |  | Studies controlling the most important factors | Studies controlling the other main factors |  | Assessment of outcome with independency | Adequacy of follow-up length | Lost to follow-up acceptable |  |
|  |  |  |  |  |  |  |  |  |  |  |  |  |
| Kharbanda (2018) | 1 | 1 | 1 | 1 |  | 1 | 0 |  | 1 | 1 | 1 | 8 |
| Scheller (2017) | 1 | 1 | 1 | 1 |  | 1 | 1 |  | 1 | 1 | 1 | 9 |
| Panagiotou (2015) | 1 | 1 | 1 | 1 |  | 1 | 1 |  | 1 | 1 | 1 | 9 |
| Lipkind (2017) | 1 | 1 | 1 | 1 |  | 1 | 1 |  | 1 | 1 | 1 | 9 |
| Baril (2015) | 1 | 0 | 1 | 1 |  | 1 | 1 |  | 1 | 1 | 1 | 8 |
| Faber (2019) | 1 | 1 | 1 | 1 |  | 1 | 0 |  | 1 | 1 | 1 | 8 |
| Bukowinski (2020) | 1 | 1 | 1 | 1 |  | 1 | 1 |  | 1 | 1 | 1 | 9 |
| Kharbanda (2021) | 1 | 1 | 1 | 1 |  | 1 | 1 |  | 1 | 1 | 1 | 9 |

| TABLE S2 Quality analysis of the included studies by modified Jadad scale. | | | | | |
| --- | --- | --- | --- | --- | --- |
| Study | Randomization | Randomization concealment | Double blind | Withdrawals and dropouts | Score |
| Angelo (2014) | 1 | 1 | 0 | 1 | 3 |
| Moreira (2016) | 1 | 1 | 0 | 1 | 3 |
| Chen (2019) | 1 | 1 | 2 | 1 | 5 |
| Garland (2009) | 1 | 1 | 2 | 1 | 5 |
