# Supplementary material for: Association of periconceptional or pregnancy exposure of HPV vaccination and adverse pregnancy outcomes: a systematic review and meta-analysis with trial sequential analysis
Source: Front Pharmacol. 2023 May 9;14:1181919. doi: 10.3389/fphar.2023.1181919 (PMC10203546; doi:10.3389/fphar.2023.1181919)

**Figure S1** Funnel plot of publication bias on spontaneous abortion in cohort studies.


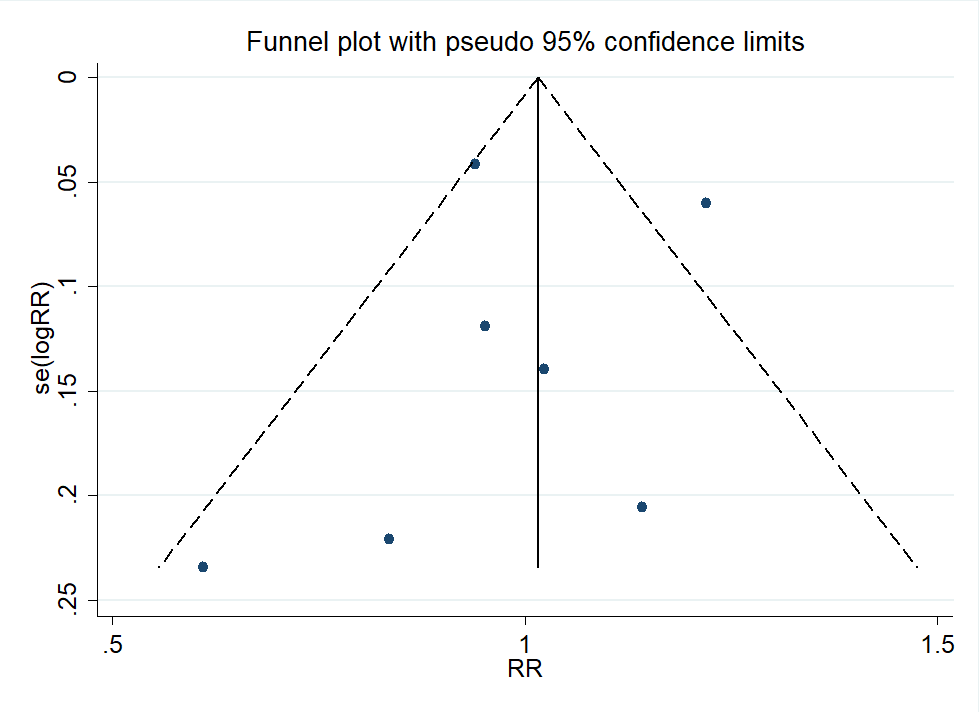


**Figure S2** Sensitivity analysis on spontaneous abortion in cohort studies.


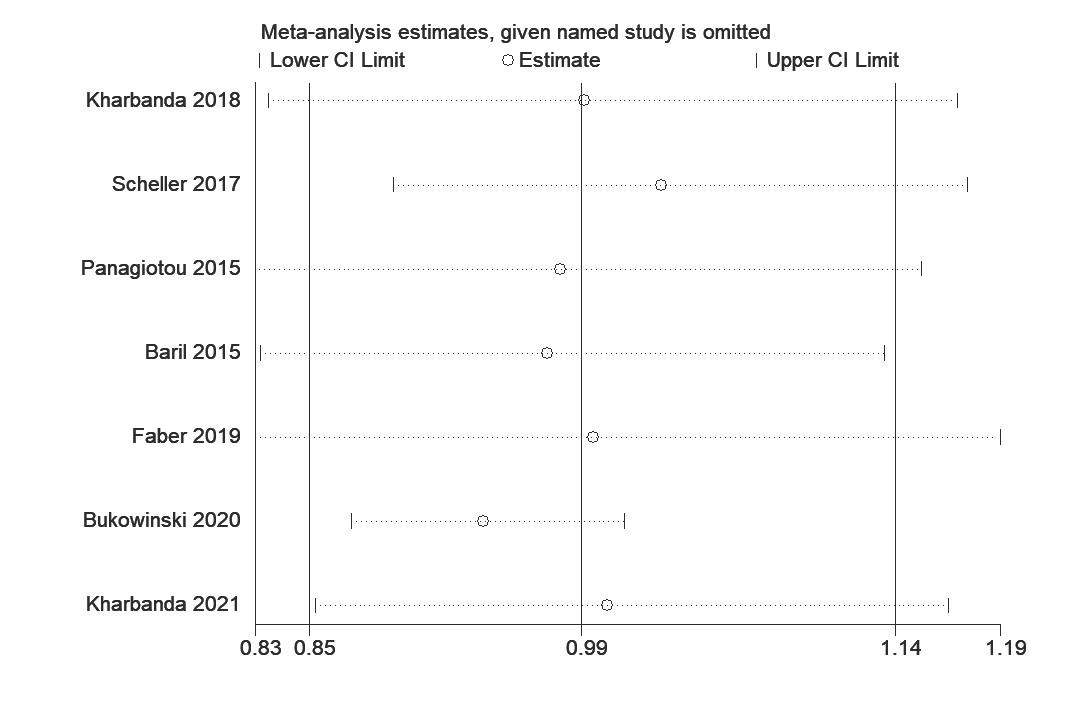

Supplement: Supplementary file 3 [file Table3.DOCX]
